# Inhibition of cathepsin X enzyme influences the immune response of THP-1 cells and dendritic cells infected with *Helicobacter pylori*

**DOI:** 10.2478/raon-2013-0043

**Published:** 2013-07-30

**Authors:** Miha Skvarc, David Stubljar, Andreja Natasa Kopitar, Samo Jeverica, Bojan Tepes, Janko Kos, Alojz Ihan

**Affiliations:** 1 University of Ljubljana, Faculty of Medicine, Institute of Microbiology and Immunology, Ljubljana, Slovenia; 2 AM - Rogaska Diagnostic Centre, Rogaska Slatina, Slovenia; 3 Faculty of Pharmacy, University of Ljubljana, Ljubljana, Slovenia; 4 Department of Biotechnology, Jožef Stefan Institute, Ljubljana, Slovenia

**Keywords:** cathepsin X, macrophages, dendritic cells, hampered immune response, toll-like receptors, cytokines

## Abstract

**Background:**

The immune response to *Helicobacter pylori* importantly determines the outcome of infection as well as the success of eradication therapy. We demonstrate the role of a cysteine protease cathepsin X in the immune response to *H. pylori* infection.

**Materials and methods:**

We analysed how the inhibition of cathepsin X influenced the immune response in experiments when THP-1 cells or dendritic cells isolated from patients were stimulated with 48 strains of *H. pylori* isolated from gastric biopsy samples of patients which had problems with the eradication of bacteria.

**Results:**

The experiments, performed with the help of a flow cytometer, showed that the expression of Toll-like receptors (TLRs), especially TLR-4 molecules, on the membranes of THP-1 cells or dendritic cells was higher when we stimulated cells with *H. pylori* together with inhibitor of cathepsin X 2F12 compared to THP-1 cells or dendritic cells stimulated with *H. pylori* only, and also in comparison with negative control samples. We also demonstrated that when we inhibited the action of cathepsin X in THP-1 cells, the concentrations of pro-inflammatory cytokines were lower than when THP-1 cell were stimulated with *H. pylori* only.

**Conclusions:**

We demonstrated that inhibition of cathepsin X influences the internalization of TLR-2 and TLR-4. TLR-2 and TLR-4 redistribution to intra-cytoplasmic compartments is hampered if cathepsin X is blocked. The beginning of a successful immune response against *H. pylori* in the case of inhibition of cathepsin X is delayed.

## Introduction

One of the important host factors that affect cure rates for *Helicobacter pylori* infections is the immune response to the infection. *H. pylori* antigens are recognised by epithelial cells, macrophages, and dendritic cells with the help of Toll-like receptors (TLRs) and Nod-like receptors (NLRs).^1^ Activation of immune cells leads to *H. pylori*– specific adaptive T helper type 1 response.^2^ In the case of impaired host immunity, a defective immune response to *H. pylori* may be a reason for *H. pylori* eradication failure.^3^ Chronic infection with *H. pylori* can also be the result of the host’s inability to induce an appropriate immune response.^4^

The discovery of cathepsin X brought new knowledge to how different proteins contribute to the immune response to *H. pylori.*^5^ Patients infected with *H. pylori* expressed more cathepsin X than the healthy control group. Cathepsin X was located in macrophages gathered from gastric mucosa. Afterwards it was discovered that patients with *H. pylori* gastritis had a higher concentration of cathepsin X protein and cathepsin X mRNA levels in gastric mucosa compared to *H. pylori* negative patients.^6^ Cathepsin X was also up regulated in the gastric mucosa of patients with gastric cancer in contrast to those without gastric cancer.^7^

Cathepsin X is a lysosomal cysteine protease found predominantly in the cells of monocyte/macrophage lineage. It acts as a monocarboxypepidase and has strict positional and narrower substrate specificity relative to other human cathepsins.^8^ Cathepsin X is capable of cleaving regulatory motifs at the C-terminus affecting the function of the β_2_ subunit of integrin receptors and alpha and gamma enolase. It was demonstrated that via activation of β_2_ integrin receptor Mac-1 (CD11b/CD18), active cathepsin X enhances the adhesion of monocytes/macrophages to fibrinogen and regulates phagocytosis.^9^ By activating the Mac-1 receptor, cathepsin X may also regulate the maturation of dendritic cells (DCs), a process that is crucial in the initiation of adaptive immunity.^10^ Cathepsin X also activates the other β_2_ integrin receptor, LFA-1 (CD11a/CD18), which is involved in the proliferation of T lymphocytes.^11^

In our study we wanted to test the hypothesis that inhibition of cathepsin X influences the successful immune response to an infection with *H. pylori.*

## Materials and methods

### Strains of *H. pylori* and patients

We analysed the influence of 40 *H. pylori* strains on the immune response of THP-1 cells. 20 strains of the bacteria were sensitive to all tested antibiotics. 20 strains of the bacteria were resistant to clarithromycin. The strains were isolated from gastric biopsies of patients with chronic gastritis. We also included a total of 8 strains of bacteria from dyspeptic patients with chronic gastritis that had problems with eradication of *H. pylori.* These patients gave 24 ml of blood that was later used to isolate DCs. Ethical approval was obtained for this study (National Medical Ethics Committee of the Republic of Slovenia).

### Isolation of *H. pylori* and antibiotic susceptibility testing

Gastric biopsy samples were inoculated onto BHI agar with 10% horse blood and checked every 3 days for the total of 12 days for the presence of colonies typical of *H. pylori* and the identity confirmed with urease, catalase and oxidase enzyme activity. Antimicrobial susceptibility was determined with an Etest (bioMerieux, France) using ISO Sensitest agar supplemented with 10% horse blood. Antimicrobial susceptibility against clarithromycin, metronidazole, amoxicillin, tetracycline, and levofloxacin was determined. Metronidazole susceptibility was additionally tested with the breakpoint agar dilution method with the metronidazole concentration of 8 mg/L. Susceptibility breakpoints used to determine resistance were in accordance with the EUCAST standards. The presence of *cagA* and v*acA* genes was determined with PCR method as stated in the previous article.^12^

The number of bacteria used in the experiment with THP-1 and DCs was determined by the absorption at A_550_, with 0.8 optical density (OD) units corresponding to 10^8^ CFU/ml of bacteria. *H. pylori* bacteria were washed three times with PBS and stored at −20°C.

### Flow cytometer analysis

All the data was collected on a FACSCanto flow cytometer and expression of different proteins was measured as MFI of various markers. We also assessed the MFI of molecules on the cell membranes using FlowJo (TreeStar, USA) and FACSDiva™ (BD Biosciences, UK) analysis software.

### Isolation and culture of THP-1 cells and DCs

The medium used throughout the experiment was Advanced RPMI 1640 supplemented with 2 mM L-glutamine, 5% FCS, and antibiotics (Hyclone, Logan, UT, USA). THP-1 cells (TIB-202™) were obtained from LGC Promochem, UK. We cultured the cells for 7 days. Cells were allowed to differentiate to monocytes/macrophages at 37°C for 24 h in a 5% CO_2_ humidified atmosphere. The flasks were washed gently with PBS to remove non-adherent cells. Adherent cells were detached with PBS containing EDTA.

Peripheral blood mononuclear cells (PBMCs) were obtained through Ficoll-Paque (PAA Laboratories Ltd., UK) gradient centrifugation. Monocyte-derived DCs were generated from CD14^+^ cells isolated by using CD14 coated immunomagnetic beads (DynabeadsFlowComp, Dynal, Norway). To differentiate dendritic cells from PBMCs we used recombinant human IL-4 (Sigma, USA) at 500 U/ml, and granulocyte-macrophage colony-stimulating factor (GM-CSF) (Sigma, USA) at 50ng/ml. We added them every 2 days before the cells were harvested on day five. Immature DCs were routinely characterized by the expression of HLA-DR, CD80, CD83, CD86, and CD14.^13^ The control of cell viability (LIVE/DEAD kit, Molecular Probes, USA) was included in the analysis.

### *In vitro* stimulation of THP-1 cells and DCs by *H. pylori*

THP-1 cells were adjusted to a final concentration of 10^6^ cells/ml and 900 μl of cell suspension was added to a 24-well plate (Corning Costar, USA). 100 μl of *H. pylori* suspension was added to each well. The experiments were performed in duplicates. In the negative control samples, the bacteria were omitted and we also added polymixin B (Sigma, USA) to block any LPS effects. In the negative control samples we also added inhibitor of cathepsin X 2F12, neutralizing cathepsin X carboxypeptidase activity to see if it has any effect on the immune response in concentration 0.5 μM.^8^ THP-1 cells were incubated in the presence of bacteria for 48 hours at 37°C. Expression of TLRs was investigated using the following antibodies: TLR-2 PE and TLR-4 FITC (e-Bioscience, UK). The 1×10^6^ of THP-1 or DCs were co-cultured for two days with defrosted *H. pylori* at a 1:10 (DCs-to-*H. pylori*) ratio. We used the patients’ *H. pylori* to stimulate their isolated DCs cells.

### Inhibition of cathepsin X and analysis of TLR-2 and TLR-4 expression

To block cathepsin X effects, we added inhibitor of cathepsin X 2F12, as previously described^14^ to THP-1 cells and DCs with *H. pylori* at the concentration of 0.5 μM. The concentration used proved to be the most appropriate in the previous experiments.^10^ The inhibitor 2F12 was added at the beginning of the experiments and again after 24 hours. Cells were grown for two days, than harvested and the expression of different molecules on the cell membrane was investigated. To control the influence of inhibitor of cathepsin X 212F, the population of un-stimulated THP-1 cells and DCs with inhibitor of cathepsin X 2F12 was analysed.

### Cytokines concentrations in the supernatant of *H. pylori* primed THP-1 cells and DCs

We collected the supernatant of THP-1 cells and DCs that were stimulated with *H. pylori* only or with *H. pylori* plus cathepsin X inhibitor 2F12. The cytokines (tumour necrosis factor (TNF)-α, interleukin (IL)-1b, IL-6, IL-8, IL-10, and IL-12p70) in the supernatants were measured with the help of BD CBA Flex Set (Becton Dickinson, USA).

### Statistical analysis

Differences between study groups were analysed for statistical significance using the unpaired Student’s *t* test, with P<0.05 taken as significant. Differences between the concentrations of cytokines of the two study groups were analysed with Mann-Whitney test, with P<0.05 taken as significant. Differences between the expression of TLRs in cathepsin X inhibited and cathepsin X non-inhibited THP-1 cells or DCs primed with *H. pylori* were analysed with Student’s *t* test and with non-parametric Wilcox Signed rank test. P<0.05 differences were taken as significant. All the calculations were done with the SPSS PASW Statistics 19 program (IBM, USA).

## Results

### *In vitro* stimulation of THP-1 cells by *H. pylori* and expression of TLR-2 and TLR-4

We first measured the expression of TLR-2 and TLR-4 on THP-1 cells after stimulating the cells with *H. pylori* with or without the addition of cathepsin X inhibitor 2F12. We did not find any differences in the expression of TLRs and cytokine production between un-stimulated THP-1 cells and un-stimulated THP-1 cells with the added inhibitor of cathepsin X 2F12.

**FIGURE 1.**
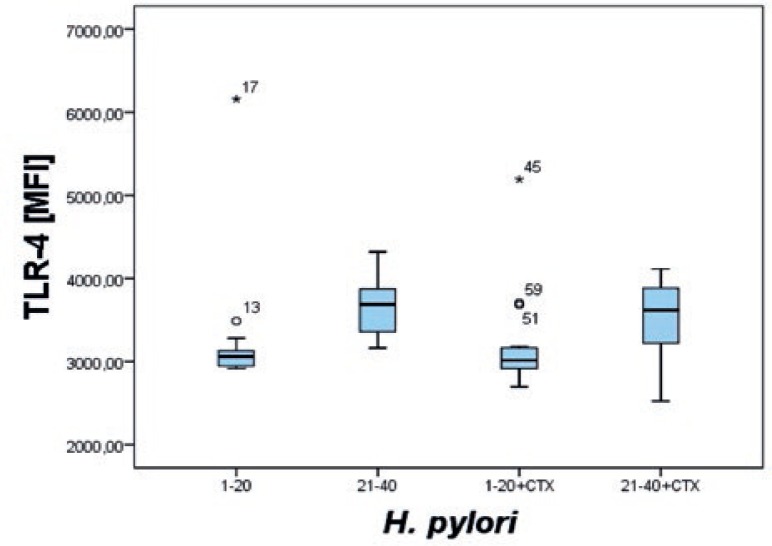
Expression of TLR-4 on THP-1 cells after stimulation with *H. pylori* or with *H. pylori* plus inhibitor of cathepsin X. 1–20 strains of *H. pylori* sensitive to clarithromycin, 21–40 strains of *H. pylori* resistant to clarithromycin, CTX inhibitor of cathepsin X.

When we compared the expression of TLRs between two groups of strains, one group being *H. pylori* strains sensitive to clarithromycin (HpS), the other strains resistant to clarithromycin (HpR), we noticed that resistant strains stimulated more THP-1 cells (% ± standard deviation-SD) to express more TLR-4 (MFI ± SD) than sensitive strains (81.51% ± 3.05 and 3656.03 MFI ± 301.05 HpR *vs*. 77.36% ± 4.85 and 3224.82 MFI ± 705.83 HpS). The difference was statistically significant (P<0.05). A similar effect was observed when we added inhibitor of cathepsin X 2F12. The TLR-4 MFI was statistically significantly higher (P<0.05) when THP-1 cells were stimulated with resistant strains (3519.33 MFI ± 428.65 HpR vs. 3172.03 MFI ± 535.67 HpS) (Figure 1).

We did not notice a statistically significant difference when we analysed the expression of TLR-2 on THP-1 cells stimulated with *H. pylori* with or without inhibitor of cathepsin X 2F12 or when we analysed the possible difference of clarithromycin sensitive or resistant strains on TLR-2.

Such a difference was not seen if we compared all strains with or without the inhibitor of cathepsin X. The mean MFI for the TLR-4 was almost the same (3440.42 ± 578.47 Hp *vs*. 3345.67 ± 510.13 Hp + inhibitor 2F12). The same was observed in the case of TLR-2 (5702.1795 ± 949.74 Hp *vs*. 5383.54 ± 741.14 Hp + inhibitor 2F12).

### Cytokines concentrations in the supernatant of *H. pylori* primed THP-1 cells

We measured the *in vitro* secretion of IL-1b, IL-12, IL-6, IL-8, TNF-α, and IL-10 by THP-1 cells primed with *H. pylori* with different susceptibility patterns and with or without inhibitor of cathepsin X 2F12. In the negative control sample (supernatants of THP–1 cells without *H. pylori* stimulation), we detected just very small concentrations of the above mentioned cytokines. The same occurred when we measured cytokines in the supernatant, where the THP-1 cells were primed only with inhibitor of cathepsin X 2F12.

**FIGURE 2.**
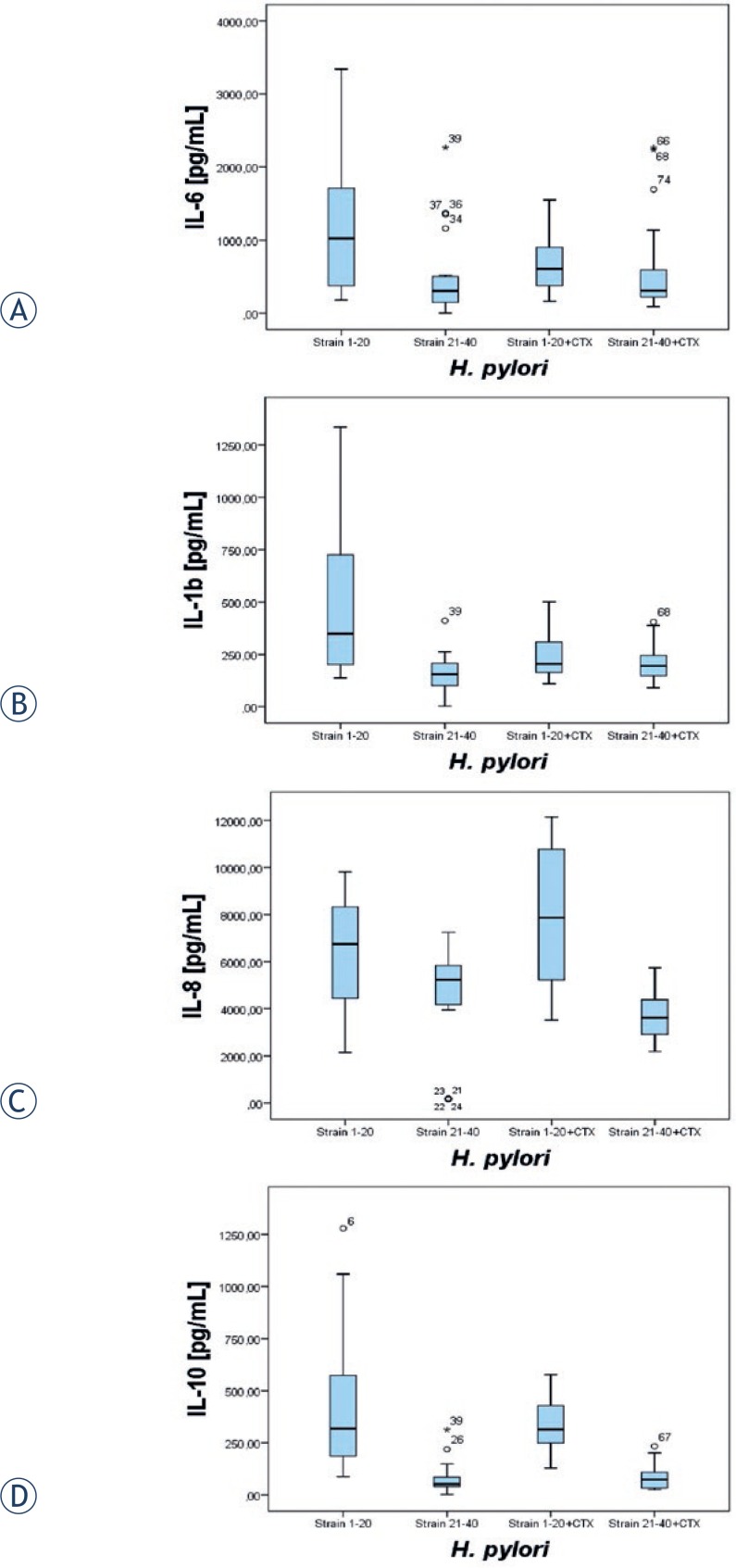
Concentrations of cytokines (pg/ml) IL-6, IL-1b, IL-8, IL-10 in the supernatant of THP-1 cells after stimulation with *H. pylori* strains or with *H. pylori* strains plus inhibitor of cathepsin X (CTX). 1–20 strains of *H. pylori* sensitive to clarithromycin, 21–40 strains of *H. pylori* resistant to clarithromycin.

After stimulation of THP-1 cells with *H. pylori* and with *H. pylori* plus inhibitor of cathepsin X 2F12, we noticed that inhibited action of cathepsin X lowers the concentration of cytokines in the group of 20 strains that are clarithromycin sensitive. Inhibition of cathepsin X influenced the cytokines IL-1b and IL-6 (P<0.01 and P<0.05) but not the others. Inhibition of cathepsin X influenced the production of IL-1b when we stimulated THP-1 cells with the group of 20 strains resistant to clarithromycin (P<0.05). The concentration of cytokines was lower in the case of the inhibited action of cathepsin X (Figure 2). The concentrations of all the other cytokines measured were lower when we added the inhibitor of cathepsin X 2F12, but did not reach statistical significance.

When we compared the concentrations of cytokines in the supernatant of THP-1 cells between 20 clarithromycin resistant and 20 sensitive strains of *H. pylori* without adding the inhibitor of cathepsin X 2F12, we noticed that clarithromycin resistant strains were weaker stimulators of cytokine production. The concentrations were statistically significantly lower for IL-6, IL-1b, IL-8, and IL-10 (P<0.01 in all comparisons). The concentrations of TNF-α and IL-12 were very low in both groups of strains, just above the limit of positivity, and were not included in the statistical analysis.

When we added the inhibitor of cathepsin X 2F12 we noticed the same phenomena between 20 clarithromycin resistant and 20 sensitive strains of *H. pylori* as in the above mentioned in the experiments without the inhibitor of cathepsin X 2F12. The concentrations of cytokines IL-8 and IL-10 (Figure 2) were again much lower where clarithromycin resistant strains plus inhibitor of cathepsin X 2F12 were used (P<0.01) in comparison to clarithromycin sensitive strains. We noticed that the biggest differences between the clarithromycin sensitive and clarithromycin resistant strains occurred in the cases of IL-1b and IL-10 where calculated F values were higher than 19.

### *In vitro* stimulation of DCs by *H. pylori* and expression of TLR-2 and TLR-4

We continued with the clinical part of the study where we wanted to know how different strains from people that had problems with the eradication of *H. pylori* influence the immune response of the DCs isolated from their blood. We first checked the phenotypic features of *H. pylori* (Table 1). All strains were sensitive to amoxicillin and tetracycline (data not shown).

For negative control we also used DCs co-cultivated with RPMI medium alone or we added an inhibitor of cathepsin X 2F12. We did not find any differences in the expression of HLA-DR and cytokine production between un-stimulated DCs and un-stimulated DCs when we added an inhibitor of cathepsin X 2F12 (data not shown).

**FIGURE 3.**
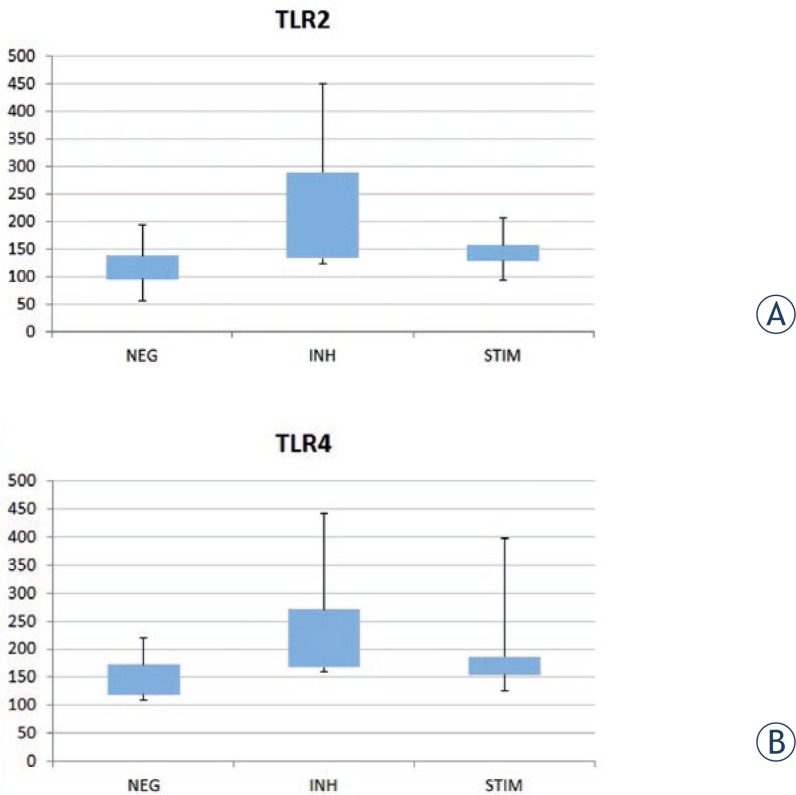
The expression of TLR-2 and TLR-4 on DCs (MFI - mean fluorescence intensity) in box plot. NEG negative control – un-stimulated DCs, INH DCs with *H. pylori* plus 2F12 - inhibition, STIM DCs with *H. pylori* - stimulation.

In the negative control sample (DCs without any stimulation) we detected a statistically significantly lower expression of TLR-4 and TLR-2 in comparison with dendritic cells stimulated with *H. pylori* only (Figure 3).

We checked the expression of TLR-2 and TLR-4 (Figure 3) on the membrane of DCs. Statistically, the expression of TLR-4 and TLR-2 presented as MFI was significantly higher (P<0.05) in the group where cathepsin X was inhibited with 2F12 (TLR-4 - *H. pylori* stimulation 161.91(146.88–232.61) *vs. H. pylori* + 2F12 inhibition 219.25 (164.38–388.83); TLR-2 - *H. pylori* stimulation 133.54(128.18–189.59) *vs. H. pylori* + 2F12 inhibition 151.045 (133.2–324.59)).

### Cytokines concentrations in the supernatant of *H. pylori* primed DCs

We measured the *in vitro* secretion of IL-12, IL-8, and IL-10 by DCs primed with *H. pylori* and with inhibitor of cathepsin X 2F12 added to *H. pylori*. In the negative control samples (supernatants of DCs without any stimulation), we did not detect any of the above mentioned cytokines. After priming DCs with *H. pylori* or with *H. pylori* + inhibitor of cathepsin X 2F12, we did not detect any statistical difference between the groups in cytokine concentrations but cytokine concentrations were lower in the dendritic cells group *H. pylori* + inhibitor of cathepsin X 2F12 - inhibition. The concentrations of TNF-α and IL-6, IL-1b detected were very low, just above the limit of positivity, and were not included in the statistical analysis.

## Discussion

For the first time, we have shown the involvement of cathepsin X in the antigen presentation with TLRs. When we stimulated THP-1 cells with different strains of *H. pylori* we noticed that the addition of the inhibitor of cathepsin X 2F12 resulted in a higher expression of TLR-4 on the membranes of THP-1 cells. This was especially true if we compared clarithromycin sensitive strains of *H. pylori* to resistant strains stimulated THP-1 cells, the resistant strains expressed more TLR-4. Our results in the clinical part of the study have shown that the expression of TLR-4 and TLR-2 was significantly higher when *H. pylori* stimulated DCs were cultivated together with cathepsin X inhibitor 2F12 compared to the dendritic cells stimulated with *H. pylori* only and also in comparison with negative control samples.

Cathepsin X is in some way responsible for the regulation of an immune response. The patients with eradication failure, despite having taken the appropriate empiric antibiotic therapy prescribed by gastroenterologist, expressed cathepsin X after stimulation with *H. pylori* antigens on the membrane of the THP-1 cell line different from the patients with successful *H pylori* eradication.^14^ How does cathepsin X influence the action of TLR-4 and TLR-2 and consequently the antigen presenting cells? *In vitro* studies have demonstrated that upon stimulation with LPS or peptidoglycan, which occurs with constant exposure to commensal bacteria, TLR-2 and TLR-4 are redistributed from the apical surface to intra-cytoplasmic compartments adjacent to the basolateral membrane.^15^ TLR-4 is redistributed to the Golgi apparatus.^16^ It was shown that during maturation, cathepsin X translocates to the plasma membrane of maturing dendritic cells, enabling Mac-1 activation and consequently cell adhesion.^10^,^14^

In our opinion, inhibition of cathepsin X hampers the internalization of TLRs, because the membrane (in the case of cathepsin X inhibition) becomes more rigid and internalization is delayed or even not possible. If cathepsin X is blocked it cannot cleave regulatory motifs at the C-terminus and this affects the function of the β2 subunit of integrin receptors of gamma enolase.^9^

We checked the influence of higher expression of TLR-4 on the membranes of THP-1 cells on the production of cytokines IL-1b, IL-8, IL-10, and IL-6, three pro-inflammatory cytokines, and one regulatory cytokine. The concentrations were lower in the group of *H. pylori* strains that were resistant to clarithromycin. The same phenomena were seen in the experiments with THP-1 cells where we added bacteria along with the inhibitor of cathepsin X 2F12. It seems that the inhibition of cathepsin X influences the concentrations of cytokines, as well on the TLRs, that are crucial for efficient regulation of immune response to *H. pylori.* We discovered that strains that are resistant to clarithromycin are less immunogenic than clarithromycin sensitive strains and that they might be capable of surviving an immune system attack for a prolonged period of time and as well develop resistance to clarithromycin that further attributes to eradication failure of *H. pylori.* In the experiments in the clinical part of the study on 8 patient’s DCs stimulated with patient’s own *H. pylori*, the difference between concentrations of cytokines between stimulation and inhibition of cathepsin X was not statistically significant. If we could increase the number of patients, we would most probably get a statistically significant difference in the concentrations of cytokines between the groups.

*H. pylori* is capable of suppressing the action of DCs and the host fails to prevent bacterial colonization. The failure to eradicate *H. pylori* during the acute phase may result in developing resistance to clarithromycin and further problems with eradication, chronic gastritis, development of peptic ulcer disease, or even gastric cancer.^17^ *H. pylori*-specific response consists of both T helper 1 and 2 subsets with high levels of IL-10-secreting T regulatory cells. It seems that *H. pylori* induces a regulatory T cell response, possibly contributing to its commensal coexistence with the human host, and that chronic gastritis and peptic ulcer disease occur when this regulatory response is inadequate.^18^ A similar effect was also seen in our study on THP-1 cells stimulated with *H. pylori.* Inhibited action of cathepsin X led to profoundly lower levels of IL-10 in the supernatant of THP-1 cells. In mice experiments *H. pylori* triggered an increase in the number of subepithelial lamina propria CD11c positive dendritic cells. Depletion of regulatory T cell numbers augmented *H. pylori*-specific effector helper T cell responses, which correlated with a lower degree of *H. pylori* colonization. Data imply that *H. pylori* targets the TLR-2 pathway to induce a regulatory T cell-skewed response.^19^ The inhibition of cathepsin X activity during dendritic cell differentiation and maturation reduced the capacity of dendritic cells to stimulate T lymphocytes.^20^ These observations are evidence that dendritic cells are involved in active editing of the immune response towards *H. pylori*. Cathepsin X may also be important in this process because some strains of *H. pylori* are able to lower the proportion of THP-1 cathepsin X positive cells. Such strains have the capability to persist in the stomach for a long time because they do not induce a strong immune response.^14^ It was presented that the impaired cellular transmigration/invasion of cells lacking cathepsin X is most likely a consequence of changes in signal transduction mechanisms leading to cell cycle delay and phenotypic changes associated with cellular senescence. It seems that high levels of cathepsin X, as observed in certain tumours, may interfere with senescence pathways and/or promote pathways engaged in proliferation, thus stimulating tumor cell growth.^21^ Recent work has suggested that defective signalling via TLR-4 may also result in an exaggerated immune response after the initial failure of innate immunity to control infection.^22^ Once inside the host cell, CagA of *H. pylori* can disrupt signalling pathways by phosphorylation-dependent and -independent mechanisms, leading to abnormal proliferation, motility, and cytoskeletal change in gastric epithelial cells.^23^ It was suggested that the eradication of CagA negative strains is harder than the eradication of CagA positive strains, because such strains are able to withstand eradication by not having important virulence factors such as CagA.^24^

We analysed the CagA/VacA status of the *H. pylori* strains in the clinical part of the study where DCs were stimulated with *H. pylori* strains. In our group of strains, CagA status is not as important for eradication success as in patients from Asia, where CagA status is relevant for the persistence of infection with *H. pylori*.

The immune response to *H. pylori* is also important for the development of gastric cancer. Recognition of pathogenic elements by TLRs and NLRs results in activation of kinases pathways and induces the synthesis and secretion of inflammatory cytokines. In the case of *H. pylori* infection, the resulting inflammation can lead to severe gastric immunopathology and cancer.^25^ In one study, cathepsin X was also linked to colorectal cancer. The researchers still think that total serum levels of cathepsin X could be a useful prognostic indicator for determining survival of patients with colorectal cancer.^26^ When the effect of inhibition of cathepsin X (also known as cathepsin Z) in mice was studied, the combined loss of cathepsin X and cathepsin B led to additive effects, resulting in significant and prominent delay of early and advanced tumour development.^27^

In conclusion the results of our study suggest that resistance to clarithromycin can be a problem for the eradication from the immune response point of view since such strains seem to be less immunogenic. We assume that the inhibition of cathepsin X to control the immune response to *H. pylori* in the cases where we have problems with eradication of *H. pylori* would not be beneficial. The immune response to infection would be delayed and this could lead to persistence of infection and possible development of resistance to antibiotics, atrophy, metaplasia and gastric cancer. On the other hand, when gastric cancer is already developed, inhibition of cathepsin X could be helpful since we could influence the process of cell senescence and also influence tumour cell growth.

## Figures and Tables

**TABLE 1.** Susceptibility of *H. pylori* strains to antibiotics and *CagA/VacA* status

**Patient’s strains**	E-test Cla/Metro/Cipro*	***CagA* status**	***VacA* status**
1	S/S/S	P	P
2	S/S/R	P	P
3	S/S/S	P	P
4	S/S/S	P	P
5	R/R/S	P	P
6	S/R/S	N	P
7	R/S/S	P	P
8	S/R/S	P	P

* Cla = clarithromycin; Metro = metronidazole; Cipro = ciprofloxacin; S = sensitive, R = resistant; P = positive; N = negative.

## References

[1] Rad R, Ballhorn W, Voland P, Eisenächer K, Mages J, Rad L (2009). Extracellular and intracellular pattern recognition receptors cooperate in the recognition of *Helicobacter pylori*. Gastroenterology.

[2] Amieva MR, El-Omar EM (2008). Host-bacterial interactions in *Helicobacter pylori* infection. Gastroenterology.

[3] Borody T, Ren Z, Pang G, Clancy R (2002). Impaired host immunity contributes to *Helicobacter pylori* eradication failure. Am J Gastroenterol.

[4] Mitchell P, Germain C, Fiori PL, Khamri W, Foster GR, Ghosh S (2007). Chronic exposure to *Helicobacter pylori* impairs dendritic cell function and inhibits Th1 development. Infect Immun.

[5] Bühling F, Peitz U, Krüger S, Küster D, Vieth M, Gebert I (2004). Cathepsins K, L, B, X and W are differentially expressed in normal and chronically inflamed gastric mucosa. Biol Chem.

[6] Krueger S, Kuester D, Bernhardt A, Wex T, Roessner A (2009). Regulation of cathepsin X overexpression in *H. pylori*-infected gastric epithelial cells and macrophages. J Pathol.

[7] Krueger S, Hundertmark T, Kalinski T, Peitz U, Wex T, Malfertheiner P (2005). Up-regulation of cathepsinX in *Helicobacter pylori* gastritis and gastric cancer. J Pathol.

[8] Kos J, Sekirnik A, Premzl A, Zavasnik Bergant V, Langerholc T, Turk B (2005). Carboxypeptidases cathepsins X and B display distinct protein profile in human cells and tissues. Exp Cell Res.

[9] Obermajer N, Premzl A, Zavasnik Bergant T, Turk B, Kos J (2006). Carboxypeptidase cathepsin X mediates beta2-integrin-dependent adhesion of differentiated U-937 cells. Exp Cell Res.

[10] Obermajer N, Svajger U, Bogyo M, Jeras M, Kos J (2008). Maturation of dendritic cells depends on proteolytic cleavage by cathepsinX. J Leuk Biol.

[11] Jevnikar Z, Obermajer N, Bogyo M, Kos J (2008). The role of cathepsin X in the migration and invasiveness of T lymphocytes. J Cell Sci.

[12] Achtman M, Azuma T, Berg DE, Ito Y, Morelli G, Pan ZJ (1999). Recombination and clonal groupings within *Helicobacter pylori* from different geographical regions. Mol Microbiol.

[13] Kopitar AN, Stegel V, Tepes B, Gubina M, Novaković S, Ihan A (2007). Specific T cell responses to *Helicobacter pylori* predict successful eradication therapy. J Infect.

[14] Obermajer N, Magister S, Kopitar AN, Tepes B, Ihan A, Kos J (2009). Cathepsin X prevents an effective immune response against *Helicobacter pylori* infection. Eur J Cell Biol.

[15] Cario E, Brown D, McKee M, Lynch-Devaney K, Gerken G, Podolsky DK (2002). Commensal-associated molecular patterns induce selective toll-like receptor-trafficking from apical membrane to cytoplasmic compartments in polarized intestinal epithelium. Am J Pathol.

[16] Hornef MW, Frisan T, Vandewalle A, Normark S, Richter-Dahlfors A (2002). Toll-like receptor 4 resides in the Golgi apparatus and colocalizes with internalized lipopolysaccharide in intestinal epithelial cells. J Exp Med.

[17] Wang X, Uto T, Sato K, Ide K, Akagi T, Okamoto M (2005). Potent activation of antigen-specific T cells by antigen-loaded nanospheres. Immunol Lett.

[18] Robinson K, Kenefeck R, Pidgeon EL, Shakib S, Patel S, Polson RJ (2008). *Helicobacter pylori*-induced peptic ulcer disease is associated with inadequate regulatory T cell responses. Gut.

[19] Zhang M, Liu M, Luther J, Kao JY (2010). *Helicobacter pylori* directs tolerogenic programming of dendritic cells. Gut Microbes.

[20] Kos J, Jevnikar Z, Obermajer N (2009). The role of cathepsin X in cell signaling. Cell Adh Migr.

[21] Kraus S, Bunsen T, Schuster S, Cichoń MA, Tacke M, Reinheckel T (2011). Cellular senescence induced by cathepsin X downregulation. Eur J Cell Biol.

[22] Higgins SC, Lavelle EC, McCann C, Keogh B, McNeela E, Byrne P (2003). Toll like receptor 4-mediated innate IL-10 activates antigen-specific regulatory T cells and confers resistance to *Bordetella pertussis* by inhibiting inflammatory pathology. J Immunol.

[23] Kurashima Y, Murata-Kamiya N, Kikuchi K, Higashi H, Azuma T, Kondo S (2008). Deregulation of beta-catenin signal by *Helicobacter pylori* CagA requires the CagA-multimerization sequence. Int J Cancer.

[24] Sugimoto M, Yamaoka Y (2009). Virulence factor genotypes of *Helicobacter pylori* affect cure rates of eradication therapy. Arch Immunol Ther Exp.

[25] Patel MK, Trombly MI, Kurt-Jones EA (2012). Innate immune responses to Helicobacter pylori infection: an overview. Methods Mol Biol.

[26] Vizin T, Christensen IJ, Nielsen HJ, Kos J (2012). Cathepsin X in serum from patients with colorectal cancer: relation to prognosis. Radiol Oncol.

[27] Sevenich L, Schurigt U, Sachse K, Gajda M, Werner F, Müller S (2010). Synergistic antitumor effects of combined cathepsin B and cathepsin Z deficiencies on breast cancer progression and metastasis in mice. Proc Natl Acad Sci U S A.

